# Oceanographic barriers to gene flow promote genetic subdivision of the tunicate *Ciona intestinalis* in a North Sea archipelago

**DOI:** 10.1007/s00227-018-3388-x

**Published:** 2018-07-11

**Authors:** Kerstin Johannesson, Anna-Karin Ring, Klara B. Johannesson, Elin Renborg, Per R. Jonsson, Jon N. Havenhand

**Affiliations:** Department of Marine Sciences, Tjärnö Marine Laboratory, University of Gothenburg, 452 96 Strömstad, Sweden

## Abstract

Pelagic larval development has the potential to connect populations over large geographic distances and prevent genetic structuring. The solitary tunicate *Ciona intestinalis* has pelagic eggs and a swimming larval stage lasting for maximum a few days, with the potential for a homogenizing gene flow over relatively large areas. In the eastern North Sea, it is found in a geomorphologically complex archipelago with a mix of fjords and open costal habitats. Here, the coastal waters are also stratified with a marked pycnocline driven by salinity and temperature differences between shallow and deep waters. We investigated the genetic structure of *C. intestinalis* in this area and compared it with oceanographic barriers to dispersal that would potentially reduce connectivity among local populations. Genetic data from 240 individuals, sampled in 2 shallow, and 4 deep-water sites, showed varying degrees of differentiation among samples (*F*_ST_ = 0.0–0.11). We found no evidence for genetic isolation by distance, but two distant deep-water sites from the open coast were genetically very similar indicating a potential for long-distance gene flow. However, samples from different depths from the same areas were clearly differentiated, and fjord samples were different from open-coast sites. A biophysical model estimating multi-generation, stepping-stone larval connectivity, and empirical data on fjord water mass retention time showed the presence of oceanographic barriers that explained the genetic structure observed. We conclude that the local pattern of oceanographic connectivity will impact on the genetic structure of *C. intestinalis* in this region.

**Electronic supplementary material:**

The online version of this article (10.1007/s00227-018-3388-x) contains supplementary material, which is available to authorized users.

## Introduction

Egg and/or larval dispersal are key components of demographic expansion and establishment in sessile and sedentary marine invertebrates. The mode and duration of larval development have been suggested to be important for population genetic structure and gene flow (Grosberg and Cunningham 2001), and hence for local adaptation (Kawecki and Ebert 2004; Kawecki 2008). Traditionally, marine invertebrate species have been considered to largely fall into one of the two groups; those with a planktonic larval stage and those without (Thorson 1946). The classical assumption has been that while the direct developers would be genetically structured down to small geographic scales, those with planktonic larvae would show much longer dispersal distances (Scheltema 1986) resulting in relatively low levels of genetic structuring. Early genetic analyses generally supported this conclusion (Burton 1983; Hedgecock 1986; Bohonak 1999; Janson 1987; Hellberg 1996). However, more recent studies using more informative genetic markers have found many exceptions to this pattern (Todd et al. 1998; Porter et al. 2002; Taylor and Hellberg 2003; Johannesson and André 2006; Weersing and Toonen 2009; Shanks 2009; Winston 2012), and mechanisms that contribute to isolation of populations with a dispersive planktonic larval stage have been suggested. These include: (1) physical barriers caused by bathymetry and spatial and temporal patterns of oceanographic currents (Galarza et al. 2009)—e.g., consistent eddy patterns and boundaries between different water masses (Werner et al. 2007); (2) morphological and behavioural characteristics—which can have an overriding influence on the extent of larval transport (Shanks and Brink 2005; Pineda et al. 2007; Shanks 2009); and (3) phenotype–environment mismatches between immigrants and the local habitat that reduce fitness of immigrants from distant habitats (Nosil et al. 2005; Marshall et al. 2010).

To learn more about the physical barriers involved in structuring marine benthic invertebrate species at a local scale, we focused on the tunicate *Ciona intestinalis*. This species has broadcast spawning and pelagic eggs and larvae. In addition, the sessile adult stage is potentially dispersive as it may attach to ship hulls and floating objects. Consequently, dispersal potential is expected to be moderate to high. The pelagic duration from spawning to larval settlement ranges from hours to a few days (Dybern 1965; Svane and Havenhand 1993). Maximum potential dispersal of gametes and larvae is, however, not only determined by these durations, but also by the strength and complexity of coastal currents. In addition, a substantial fraction of eggs, embryos, and larvae may be released and constrained within adhesive mucus strings that disperse over relatively short distances, and may contribute disproportionately to local recruitment (Svane and Havenhand 1993).

Here, we investigated connectivity among samples of *C. intestinalis* from a relatively small geographic area with sampled sites 2–110-km apart. We combined a population genetic analysis using microsatellite markers with a hydrodynamic model that quantified maximum dispersal of passively distributed eggs and larvae under the local physical conditions of ocean currents and coastal topography.

## Materials and methods

### Systematics of *C. intestinalis*

Recent taxonomic studies of *Ciona intestinalis* have identified four sibling species, of which one (“spB”) has a North Atlantic distribution (Suzuki et al. 2005; Caputi et al. 2007). This species is the only species known to be established in the southern and western Baltic Sea, the North Sea, and the English Channel, and it is also present along the eastern coast of North America (Dybern 1965; Roux et al. 2013). It is distinct from the former *Ciona intestinalis* spA now named *C. robusta* (Brunetti et al. 2015).

### Study area

**Fig. 1:**
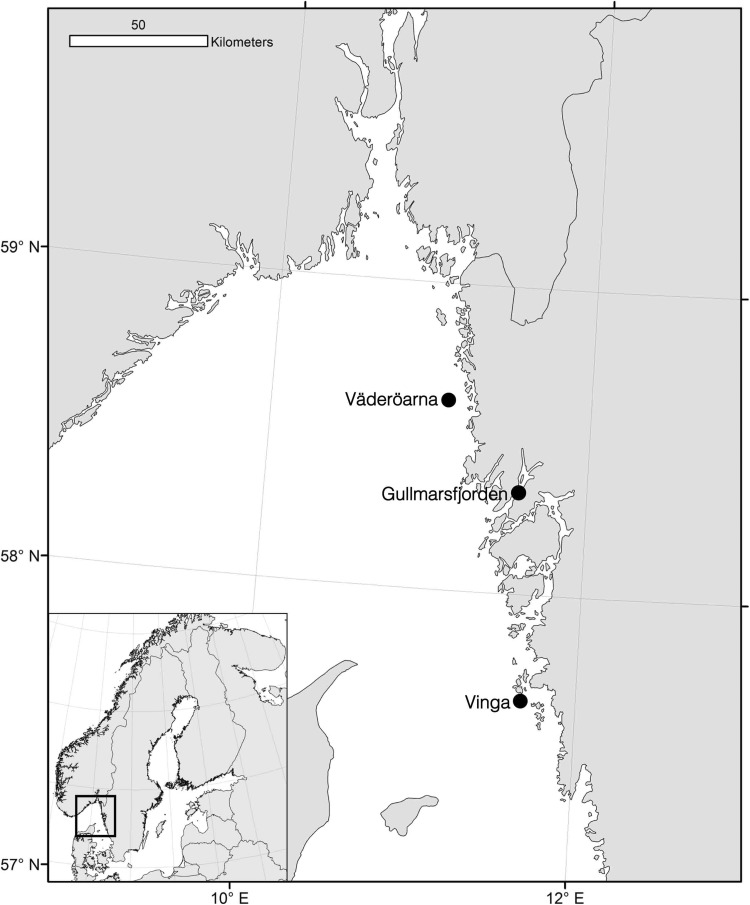
Map of the Swedish west coast indicating sampling areas. One-to-three samples were taken in each of the three sampling areas (Väderöarna, VÄD; Gullmarsfjorden, GUL; Vinga, VIN), see details in Table 1

The northern part of the Swedish west coast has an extensive archipelago with hundreds of rocky islands scattered along the coast. The islands of Väderöarna and Vinga are at the western frontier, facing the open ocean (Fig. 1). Around these islands are both deep (> 40–100 m) and shallow (< 10 m) waters, and bottom substrata are a mixture of fine sediments, sand, and rocky outcrops. The coast has several fjords, the largest and deepest of which is Gullmarsfjorden, a 1–3-km wide water body that cuts 25 km into the mainland. The maximum depth of Gullmarsfjorden is 125 m, but an entrance sill of only 45-m depth restricts exchange of water with the open sea.

This part of the North Sea is almost a tidal with spring tides ranging from 24 cm (Vinga) to 37 cm (Väderöarna) and correspondingly negligible tidal currents. The water column in the region is strongly stratified with a pycnocline at 10–15 m (depending on location, season, and weather). The origin of the surface-water layer is the Baltic Sea, but in the study area, it has a salinity of 15–25 psu and flow northward at ~ 0.5 m s^−1^. Below, the pycnocline is water of more oceanic origin that has a higher salinity (> 30 psu) and, often, a slower southward return current.

### Sampling

We sampled 40 individuals of *C. intestinalis* from each of 6 different sites distributed over three different areas (Väderöarna, Gullmarsfjorden, and Vinga, Fig. 1). In Gullmarsfjorden and Väderöarna, samples were taken from both shallow (< 5 m) and deep (> 20 m) rock substrata, usually on vertical or overhanging rock walls. In Vinga, we were only able to find *C. intestinalis* at a deep site (Table 1). To check for temporal variation, we re-sampled the deep Gullmarsfjorden in two successive years (i.e. the “sites GUL-deep11” and “GUL-deep12” are from essentially the same place, but sampled 2011 and 2012, respectively, Table 1). In this region, sampling *C. intestinalis* does not require permission from any local or national authority, and the sampled species is neither classified as endangered nor is it under any protection.

**Table 1:** Details of sampling of *Ciona intestinalis* in three areas at the Swedish west coast (see Fig. 1)

Sample	Name of locality	Coordinates	Sampling depth (m)	Date of sampling	*N*
VÄD-shall	Väderöarna, Södra Ärholmen	N 58°32′55″, E 11°5′37″	3–4	Aug. 2011	40
VÄD-deep	Väderöarna, Storön	N 58° 34′59″, E 11°4′27″	22	Aug. 2011	40
GUL-shall	Gullmarsfjorden, Jordfall	N 58°19′47″, O 11°34′15″	5–6	Aug. 2011	40
GUL-deep11	Gullmarsfjorden, Gåsklåvan	N 58° 18′36″, E 11°32′21″	27	Aug. 2011	40
GUL-deep12	Gullmarsfjorden, Gåsklåvan	N 58°18′36″, E 11°32′21″	22–28	July–Aug. 2012	40
VIN-deep	SE Vinga	N 57°37′17″, E 11°38′9″	23–25	July–Aug. 2012	40

### Genetic analyses

Genomic DNA was isolated from ethanol-preserved tissue using NucleoSpin Tissue Kit (Macherey–Nagel). Samples were genotyped at six microsatellite loci Cin-1, Cin-10 following (Andreakis et al. 2007) and Cin-12, Cin-13, Cin-15, Cin-16 following (Zhan et al. 2010). PCRs were performed with the tail method described in (Schuelke 2000), and all forward primers were 5′ modified by adding the Tail 2 sequence from (Real et al. 2009). Consequently, the Tail 2 sequence was used as the dye-labeled oligo for each primer pair. PCR products were poolplexed and separated on a Beckman-Coulter CEQ 8000 automated sequencer, and alleles were sized using the Fragment Analysis Software (Beckman-Coulter). *F*-statistics and tests for departure from Hardy–Weinberg were performed using GENEPOP 4.2 (Raymond and Rousset 1995), and we used *F*_IS_ and *F*_ST_ to infer levels of inbreeding and population differentiation, respectively. A Mantel test was used to compare the matrices of genetic and geographic distances among populations (XLSTAT 2013.6.03 version of Excel). We used individual Bayesian assignment analysis (Structure 2.2; Pritchard et al. 2000) to describe patterns of population differentiation with 5 independent runs using 50,000 burn-in steps and 500,000 length runs, each with 3 iterations. The structure analysis infers individual ancestry by assigning sampled individuals into *K* populations that minimize genotypic disequilibrium under the assumption of random mating. The *Q*-matrices obtained from structure were used in CLUMPAK (Kopelman et al. 2015) to compare and sum-up the different structure outputs while estimating the most likely value of *K* clusters, as well, according to Evanno et al. (2005). The output presented here is the result of this final analysis based on the estimated most likely *K* (see Supplementary material for estimate of optimal *K*).

We also used an assignment test in GENECLASS2 (Piry et al. 2004) to estimate the probabilities of individuals being correctly assigned to each of the sampled populations.

### Biophysical model

We used biophysical modeling to estimate levels of connectivity among study sites due to dispersal of planktonic *C. intestinalis* embryos and larvae. An oceanographic circulation model first produced spatio-temporal fields of water velocity, and an off-line trajectory model, parameterized with biological data, then calculated a large number of individual dispersal paths based on the velocity field. The local ocean circulation was modeled with the NEMO-Nordic model (Hordoir et al. 2013), which is a regional Baltic/North Sea configuration of the NEMO-ocean model (Madec 2016). Spatial resolution is 3.7 km in the horizontal dimension, and the model has 56 levels in the vertical, ranging from 3-m intervals at the surface to 22 m in the deepest parts. The NEMO-Nordic model has a free surface and the grid boxes can stretch and shrink vertically to model the tides. For a more detailed description of the model and some validation results, see (Hordoir et al. 2013).

Dispersal of *Ciona* larvae in the study area was calculated with the Lagrangian trajectory model TRACMASS (De Vries and Döös 2001). TRACMASS is an off-line particle-tracking model that calculated the transport of particles using velocity field data from the NEMO-Nordic model. Velocity fields were updated every 3 h and the trajectory calculations were done with a 15-min time step. We parameterized the trajectory modeling with available information for *Ciona* larvae. We used a 5-day pelagic larval duration which represents an upper bound for the duration of drifting eggs and larvae, although up to 6 days of larval duration has been observed (Svane and Havenhand 1993). Larval dispersal of the shallow-water populations was modeled between May and September, while dispersal of the deeper populations was modeled between June and July; this difference is in accordance with reported timing of spawning (Dybern 1965) and was used to improve the predictions from the model as much as possible. Dispersal trajectories were simulated in two depth intervals, 0–12 and 24–26 m, where 0–10 m is the most likely interval for *Ciona* larvae (Dybern 1965), although this is highly uncertain, as is drift depth for most invertebrate larvae (Corell et al. 2012). We repeated the dispersal simulations for 8 years (1995–2002) that sampled both positive and negative values of the NAO climatic cycle index (Berglund et al. 2012), which influences ocean circulation in the area. From each study site, a total of 19,600 virtual larvae per depth interval were released. Dispersal probabilities between the study sites were estimated by calculating the proportion of released particles from site *i* that ended up in site *j*. We also calculated the multi-generation connectivity based on stepping-stone dispersal over 32 single-generation dispersal events summed over all possible dispersal routes (White et al. 2010). Such multi-generation connectivity may be used to infer the long-term connectivity between populations and exploring barriers to gene flow. This is achieved by multiplying the single-generation connectivity matrix with itself n times, (in this case *n* = 32), which should be sufficient to span the ca 200-km model domain. Since the studied geographic area is highly open, there is a significant loss of larvae to areas outside the model domain making the connectivity approach zero as the number of matrix multiplications increases. We thus only interpreted the relative magnitude of multi-generation connectivity between the three study areas (Väderöarna, Gullmarsfjord, and Vinga, Fig. 1). Due to insufficient horizontal resolution of the oceanographic model (3.7 km), it was not possible to model dispersal of the populations within the narrow Gullmarsfjorden. Instead, we selected the entrance area of the fjord outside the sill to represent these populations in the trajectory model. To obtain an estimate of the exchange of larvae from inside the Gullmarsfjorden with the coastal water outside the fjord, we used information from Arneborg (2004) who, from empirical data, calculated an average turnover time above the pycnocline of 16–26 and 40 days below the pycnocline.

## Results

### Genetic variation within samples

All samples contained similarly high amounts of genetic variation with an average of 11 alleles per locus and expected levels of heterozygosity around 0.75 (Table 2, and see supplementary file; S1 showing the genotypes of all 240 individuals). Notably, observed levels of heterozygosity were roughly 20% lower than expected values, and mean inbreeding coefficients (*F*_IS_) strongly positive, indicating deficiency of heterozygote individuals in all samples. However, individual loci showed very different patterns with two loci (Cin-10 and Cin-15) showing significant *F*_IS_ values in all samples, whereas the remaining four loci showed few significant *F*_IS_ values (Table 2). Observed heterozygosities were also much higher and approached expected values in the four loci with mostly non-significant *F*_IS_ (Table 2).

**Table 2:** Genetic variation within samples of *Ciona intestinalis*

Sample	Mean no of alleles	*H*_obs_	*H*_exp_	*F*_IS_						
				Cin-10B	Cin-12B	Cin-16B	Cin-1	Cin-13	Cin-15	Average over all loci
VÄD-shall	10	0.55	0.75	**0.55**	0.14	− 0.02	0.13	0.15	**0.78**	0.29
VÄD-deep	13	0.51	0.78	**0.76**	0.14	0.15	**0.16**	0.16	**0.70**	0.34
GUL-shall	10	0.51	0.76	**0.70**	0.04	0.35	0.18	0.30	**0.44**	0.33
GUL-deep11	10	0.49	0.70	**0.53**	0.02	**0.51**	0.05	**0.26**	**0.65**	0.34
GUL-deep12	10	0.56	0.71	**0.54**	0.14	0.17	0.02	0.15	**0.42**	0.24
VIN-deep	12	0.52	0.79	**0.54**	− 0.05	**0.35**	**0.29**	0.18	**0.78**	0.35
Average over all sites	11	0.52	0.75	0.60	0.07	0.25	0.14	0.20	0.63	0.32
Mean no of alleles				8	11	11	12	13	9	
Mean *H*_obs_				0.22	0.77	0.50	0.68	0.67	0.29	
Mean *H*_exp_				0.58	0.83	0.68	0.79	0.83	0.78	

Mean number of alleles per locus, expected and observed heterozygosity, and inbreeding coefficient *F*_IS_ (shown for each locus, and averaged over loci). *N* = 40 individuals in all samples. *F*_IS_ is calculated using the method described in (Weir and Cockerham 1984). Bold figures indicate statistically significant (*p* < 0.05) deviation from Hardy–Weinberg after sequential Bonferroni correction following (Rice 1989)

### Genetic differentiation among samples

The samples of *C. intestinalis* were generally highly differentiated with a majority of *F*_ST_ values remaining statistically significant after Bonferroni correction (Table 3). Conversely, two of the pairwise comparisons were largely undifferentiated: the two Gullmarsfjorden deep samples taken in two consecutive years at the same site (GUL-deep 11 and GUL-deep 12) were not significantly different (*F*_ST_ = 0.002, Table 3) suggesting temporal stability over these years at this site. Furthermore, the two open-coast deep-water samples (VÄD and VIN) were also genetically very similar (*F*_ST_ ≈ 0, Table 3) despite these localities being ~ 110-km apart (Fig. 1). All other pairwise comparisons showed moderate-to-large levels of differentiation (*F*_ST_ = 0.05–0.12, Table 3). For example, samples separated by only 2–4 km but from different depth layers (shallow and deep sites) were substantially genetically differentiated (GUL-shall and GUL-deep11, and VÄD-shall and VÄD-deep, Table 3).

**Table 3:** Genetic differentiation among samples of *Ciona intestinalis*

Sample	VÄD-shall	VÄD-deep	GUL-shall	GUL-deep11	GUL-deep12	VIN-deep
VÄD-shall	x	3.7	1.9	2.1	2.0	3.5
VÄD-deep	**0.063**	x	3.6	4.3	4.7	N.A.
GUL-shall	**0.116**	**0.066**	x	1.9	2.0	3.9
GUL-deep11	**0.104**	**0.055**	**0.116**	x	156.0	4.6
GUL-deep12	**0.111**	**0.051**	**0.113**	0.002	x	4.7
VIN-deep	**0.067**	0.000	**0.061**	**0.052**	**0.050**	x

Below diagonal is pairwise *F*_ST_ estimates, and above diagonal is estimates of effective migration (*Nm*). Figures in bold are significant at *p *< 0.05 after sequential Bonferroni correction following (Rice 1989)

Under the assumption of selectively neutral genetic variation and a two-dimensional distribution of populations (island model), the range of genetic differentiation found among the latter group of samples corresponded to levels of effective migration (*Nm*) of only 2–5 individuals per generation (Table 3). *F*_ST_ estimates for the different microsatellite loci were similar when averaged over all samples (see supplementary file; S2, Table S1 showing average *F*_ST_ among samples per locus), which suggests that none of the loci were affected by strong differential selection, and hence, the *Nm* estimates are likely to represent gene flow. However, we found no support for isolation by distance (see supplementary file; S2, Fig. S1 showing *F*_ST_ as a function of geographic distance and the result of a Mantel test), and hence, gene flow was not simply an effect of the geographic distances among sites. These results, instead, suggested a presence of local barriers to gene flow between some of the sites.

**Fig. 2:**
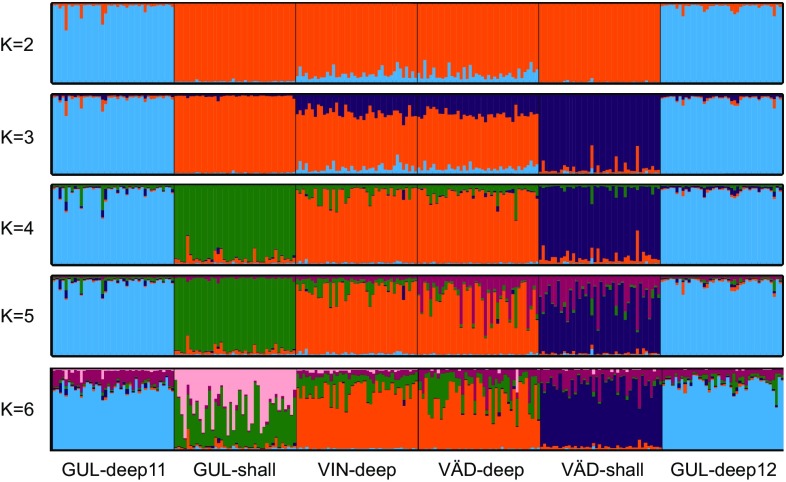
Population genetic structure of *C. intestinalis* on the Swedish west coast as revealed by structure analyses based on five microsatellites, and in which individuals are assigned to one of the *K* clusters. The optimal number of clusters was *K* = 4

The structure analysis showed support for the presence of local barriers with strong overall support for a similar genetic structure as indicated by the pairwise *F*_ST_ estimates, that is, the two deep-water samples (VÄD-deep and VIN-deep) grouped, and the two samples from GUL-deep sampled in different years made a separate group, while both shallow-water samples were different from each other and from all the deep-water samples at the *K* = 4 (Fig. 2). Note that with all loci included in the analysis, the optimal number of clusters (*K*) was 3. However, one of the loci (Cin-15) had a large number of missing data (presumably individuals homozygote for a null allele, see “Discussion”) and removing this locus resulted in *K* = 4 being the optimum (see supplementary file; S2, Fig. S2 showing estimation of optimal *K*). Importantly, the overall result of the structure analysis was the same independent of 5 or 6 loci, and the only difference was in the optimal value of *K*. We show the analysis for 5 loci in Fig. 2.

**Fig. 3:**
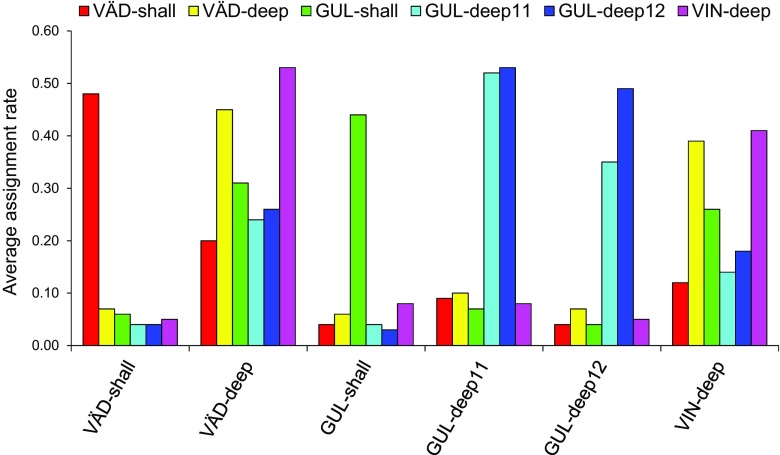
Average assignment rate of individual genotypes to the six populations estimated as average probabilities of individuals being correctly assigned to each of the sampled populations using the GENECLASS2 assignment test

The results of the assignment test showed that some of the samples were clearly more discrete than others with high levels of self-assignments, for example, the shallow-water fjord site (GUL-shall) and the open-coast shallow-water sample (VÄD-shall) (Fig. 3). The samples from the deep fjord site taken in two consecutive years (GUL-deep 11 and GUL-deep 12) showed a high degree of assignment to each other. In contrast, individuals from the two open-coast deep-water sites (VIN-deep and VÄD-deep) had relatively high proportions of assigned individuals originating in other sites (Fig. 3).

### Connectivity modeling

**Fig. 4:**
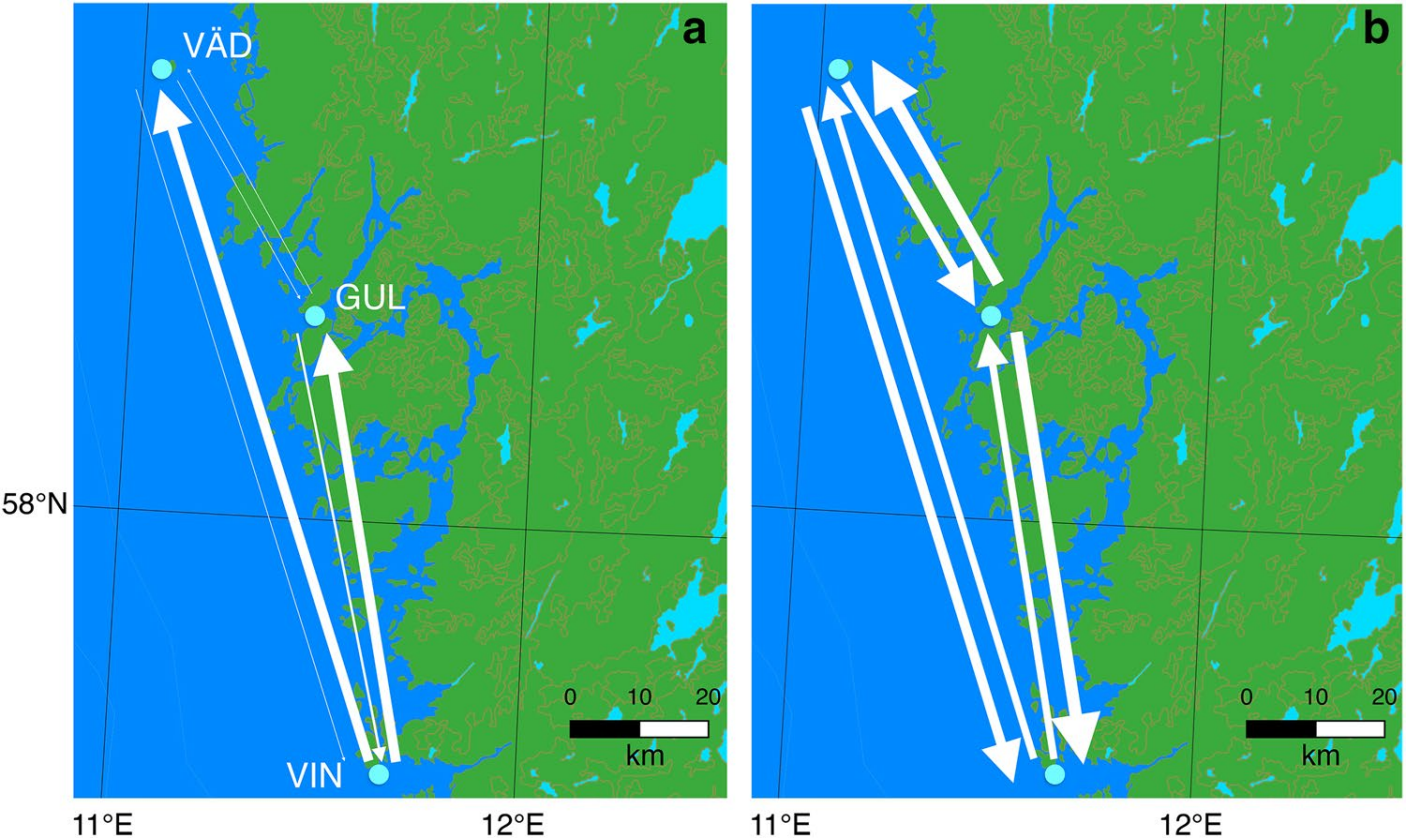
Biophysical modeling of connectivity among study populations assuming passive larval transport of eggs and larvae during 5 days. The connectivity that results after 32 successive generations of dispersal is illustrated. Arrow thickness is proportional to magnitude of connectivity on a logarithmic scale. Model simulations were run at  two different depths; larval depth 0–12m and habitat depth down to15m (**a**), and larval depth 24–26m and habitat depth deeper than 15m (**b**)

The biophysical model simulated passive dispersal of eggs and larvae, and multi-generational connectivity during 32 consecutive generations under the assumption of a maximum pelagic larval duration of 5 days. The results showed that from the surface down to a depth of 12 m, passive dispersal should be expected in a mainly northward direction with larvae originating in the southernmost site (VIN) spreading north (Fig. 4a, and see supplementary file; S2, Table S2 showing relative connectivity among all sites included in the biophysical model). At a depth of 24–26 m, however, the strength and direction of currents were equivocal, indicating that northbound and southbound larval flows along the coast are about equally likely and that spread of larvae from inner coastal sites to open-coast sites is more likely than spread in the opposite direction (Fig. 4b). Importantly, due to the grid size of the model, it was not possible to include the two fjord sites directly in the model, and thus, the inner coastal site of the model was a location outside the sill of the Gullmarsfjorden. The implications of this choice and the influence of water retention times in the Gullmarsfjorden are considered below.

## Discussion

Genetic variation within our samples was generally large, with levels of expected heterozygosity around 75%. This is high but similar to earlier studies of *C. intestinalis* (Zhan et al. 2012; Hudson et al. 2016). Further corroborating earlier studies, we also found strong heterozygote deficiencies at some loci. Heterozygote deficiency has several alternative explanations: (1) a Wahlund effect from mixing of populations; (2) selection against heterozygotes; (3) inbreeding or selfing; and (4) null alleles or large allele drop-outs. We rejected all of these explanations except that of null alleles for the following reasons: A Wahlund effect may arise from the mixing of larvae from genetically distinct populations. To test this, we removed individuals that were not strongly assigned to the sample of origin and recalculated inbreeding coefficients. This did not remove heterozygote deficiencies, but increased the overall *F*_IS_ by 2%. Selection against heterozygotes seemed unlikely as microsatellites are in non-coding regions of the genome and selection against heterozygotes would only be possible if a microsatellite locus was closely linked to a locus under selection. *Ciona intestinalis* is a simultaneous hermaphrodite and selfing is possible, but reported to be low or absent due to barriers to selfing (Byrd and Lambert 2000; Harada et al. 2008; Saito et al. 2012; Philippi and Yund 2017). However, the observed heterozygote deficiency in one of the loci (Cin-15) seemed likely to be due to null alleles, as a large proportion of individuals (24%) did not give any PCR product and were presumably homozygotes for one or more null alleles (see supplementary file; S2, Table S3 showing missing data and number of sites for which MICRO-CHECKER suggested null alleles). In the other locus with heterozygote deficiency (Cin-10), only 6 individuals (of 240) lacked PCR products, and the presence of null alleles was considered as less likely.

Although the present study was undertaken in a relatively small geographic area, we found *C. intestinalis* to be strongly genetically structured. The structuring, however, did not reflect geographic distances among samples. Even so, the species has a pelagic larva the swimming speed of a larva is only a few percent, or less, of the typical speed of the oceanographic currents in the area (Havenhand 1991). Consequently, directions and speeds of local currents are more likely than geographic distance to determine the genetic structure of the species. A strong support for this suggestion was that the two geographically most distant samples were genetically very similar (deep VÄD and deep VIN). In contrast, samples from the fjord (Gullmarsfjorden) appeared clearly different from the open-coast samples, and samples from surface waters were genetically different from samples from below the pycnocline. Overall levels of genetic differentiation in our study were consistent with an earlier study in which *C. intestinalis* was sampled at similar geographic scales (10–100 km) on the Atlantic coast of North America (Zhan et al. 2010). A study by Hudson et al. (2016) investigating the population structure of *C. intestinalis* in the English Channel, an area of intense human activity and considerable potential for human-aided transport of tunicate larvae, also showed similar levels of genetic differentiation. In this study, most *C. intestinalis* populations were rather similar, but a few populations (geographically close to other populations) stood out as genetically different from the rest. The authors suggested a mix of anthropogenic and natural causes explaining the complex population genetic structure of *C. intestinalis* in the English channel.

Notably, the magnitude of genetic differentiation we observed among nearby sites (*F*_ST_ ranging from 0.054 to 0.093) was similar to what has been observed in marine species with little or no dispersal capacity (Coyer et al. 2003; Tatarenkov et al. 2007; Mäkinen et al. 2008). Such strong differentiation can occur in markers linked to genes under selection (Larsson et al. 2007); however, it is highly unlikely that the strong differentiation we observed consistently over all six loci was the result of selection on all these loci. A more probable explanation is that these patterns were caused by barriers to gene flow among populations or, possibly, by a complex demographic history of the populations. Another possibility would be that larvae are more or less completely retained in the mucus (although reported rates of retention are in the range 40–60%, Svane and Havenhand 1993). However, the very high genetic similarity between the two deep-water sites (VIN and VÄD) 110-km apart is difficult to explain under the assumption that practically, all larvae are trapped in the mucus. Moreover, human-aided migration (adults attached to ship hulls) would be much more likely to occur between the two shallow-water sites (VÄD-shall and GUL-shall) than between deep-water sites, but we found genetic differentiation between the shallow sites while not between the two deep, open-coast sites (VIN-deep and VÄD-deep).

On the other hand, our biophysical model showed that connectivity between the two deep open-coast sites may be relatively strong. The reason for this is that in addition to deep-water currents in both directions, there are extensive deep-water benthic surfaces available for colonization of *C. intestinalis*. Indeed, it seems possible that recruits to the deep populations of both VIN and VÄD derive from a common extensive open-coast, deep-water population of *C. intestinalis*. Another possibility is that deep-water larvae migrate to surface waters and get transported by the northbound surface current before they migrate back to deep water and settle. Such a hypothesis is supported by observations of *Ciona* larvae being most frequent in the upper water layers in this area (Dybern 1965), although studies of larvae of other tunicate species suggest that this complex behaviour is unlikely (see below).

The spatial resolution of our biophysical model did not permit accurate representation of the hydrodynamics of the Gullmarsfjorden, and we, therefore, assessed connectivity to a site immediately outside the sill of the Gullmarsfjorden. Water residence times in the fjord, inside the sill, are 16–26 days for surface water and 40 days for deep water (Arneborg 2004). Even the shortest of these periods is considerably longer than the maximum pelagic period of eggs and larvae of *C. intestinalis* (7 days in total, Svane and Havenhand 1993). Thus, connectivity between *C. intestinalis* open-coast and fjord populations would be strongly constrained by low dispersal across the fjord sill. Indeed, our genetic data show significant differentiation between *C. intestinalis* from both shallow and deep fjord sites and all sites outside the fjord (Table 3).

In the two locations from which we had both shallow and deep samples (GUL and VÄD), genetic differences indicated significant barriers to gene flow between surface and deep populations (*F*_ST_ = 0.11 in GUL and *F*_ST_ = 0.066 in VÄD). In the study region, weak tidal currents and a strong northbound surface current bringing low-salinity water from the Baltic Sea create strong stratification with a low-salinity surface-water layer ~ 10–15-m deep above a more oceanic deep-water layer. The strong density pycnocline that separates these two layers constitutes an effective barrier to passively dispersing particles, and even to actively swimming larvae (Sameoto and Metaxas 2008; Daigle and Metaxas 2011), including tunicate tadpoles (Vázquez and Young 1996). Moreover, freely released eggs are slightly adhesive and may often remain attached to the substratum either singly or in mucus strings (Svane and Havenhand 1993), further reducing dispersal. Thus, it seems likely that a combination of restricted dispersal due to adhesive eggs, spawning in mucus strings, and/or restricted vertical transport of larvae over the pycnocline impedes gene flow between *C. intestinalis* living in shallow- and deep-water masses from the same areas.

In summary, the oceanographic barriers caused by the fjord sill and by the pycnocline between deep- and shallow-water bodies, together result in a subdivision of *C. intestinalis* in the study area into different genetic populations: separate deep- and surface-water populations inside as well as outside the fjord, and also different populations inside and outside the fjord even when coming from similar depths (Fig. 3). An intriguing effect of such a differentiation/isolation between surface- and deep-water populations is that native deep-water populations may be less affected by admixture caused by human translocations, such as shipping, compared to the shallow-water populations.

Barriers to gene flow between populations can create strong opportunities for local adaptation in various traits (Kawecki and Ebert 2004). Local adaptation to different salinities and temperatures, for example, is common among marine organisms and in particular in species that are genetically structured and have populations established in different habitats (Marshall et al. 2010; Sanford and Kelly 2011). Notably, earlier investigations of *C. intestinalis* in the Gullmarsfjorden describe a deep-water population that spawns once a year in late spring and a surface-water population that spawns twice during spring–summer (Dybern 1965). Individuals of the deep-water population, furthermore, grow to a larger size than do individuals of the shallow-water population (Dybern 1965; Svane 1983). Whether these differences are evidence of local adaptation (i.e. inherited differences), or the result of induced responses to different salinities, temperatures, and food availabilities remains untested, however. In a recent study, larval development in different salinities was compared between individuals from surface and deep-water populations of *C. intestinalis* from the Gullmarsfjorden and Väderöarna (Renborg et al. 2014). That study found substantial plasticity in larval salinity tolerance—optimal salinities for larval development carefully matched the salinities to which parents had been acclimatized for ~ 2 weeks prior to spawning, and these responses were independent of adult origin (Renborg et al. 2014). It seems likely, then, that local adaptation to different salinities during early life-history stages has not evolved in these populations. Intriguingly, however, the transgenerational effects (parental salinity experience influencing larval salinity tolerance norms, Renborg et al. 2014) will tend to impede connectivity between populations in different habitats, because fitness of any larvae that do cross salinity gradients will be reduced. This “phenotype–environment” mismatch has been argued to be an important component of isolation in marine organisms (Marshall et al. 2010), and may further drive the development of locally adapted behavioural habitat preferences in larvae of this species.

## Conclusion

In the benthic marine invasive tunicate *C. intestinalis,* we observed striking genetic subdivision of a coastal population despite strong potential for gene flow through pelagic larvae. Moreover, the observed genetic subdivision was not an isolation-by-distance effect. Using biophysical modeling, we found oceanographic barriers to dispersal that could explain the genetic subdivision. These barriers included a fjord sill, which impeded gene flow between fjord and open-coast sites, and a strong pycnocline, which constrained larval dispersal and gene flow between surface and deep-water masses. Our results show that small-scale coastal oceanographic barriers may be important mechanisms shaping the genetic structure of marine populations.

## Electronic supplementary material

Below is the link to the electronic supplementary material.

**Supplementary file S1:** Genotypes of all 240 individuals of *Ciona intestinalis* (XLSX 69 kb)

**Supplementary file S2:** This file contains 2 figures and 3 tables (PDF 130 kb)
