# Supplementary material for: Oceanographic barriers to gene flow promote genetic subdivision of the tunicate Ciona intestinalis in a North Sea archipelago
Source: Mar Biol. 2018 Jul 11;165(8):126. doi: 10.1007/s00227-018-3388-x (PMC6061499; doi:10.1007/s00227-018-3388-x)
Supplement: Supplementary file 2 — Supplementary file S2: This file contains 2 figures and 3 tables (PDF 130 kb) [file 227_2018_3388_MOESM2_ESM.pdf]

Supplementary file 2.

Oceanographic barriers to gene flow promote genetic subdivision of the tunicate *Ciona intestinalis* in a North Sea archipelago

Kerstin Johannesson*, Anna-Karin Ring, Klara B Johannesson, Elin Renborg, Per R Jonsson, and Jon N Havenhand

Fig. S1: Genetic differentiation (F_{ST}) as a function of geographic distance and the result of a Mantel test.

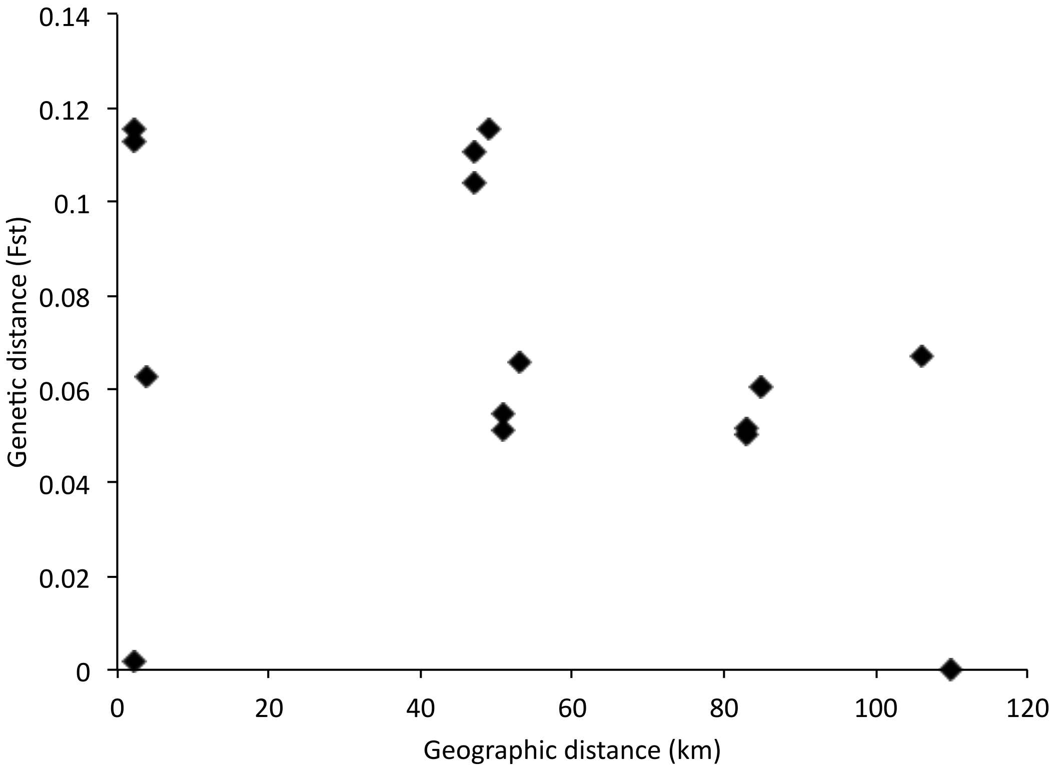

A Mantel test shows a non-significant correlation between the two matrices, the genetic differentiation matrix and the geographic distance matrix (r = -0.35, p = 0.20, Mantel test).

Fig. S2: The optimal number of clusters (K) estimated following Evanno et al. (2005).

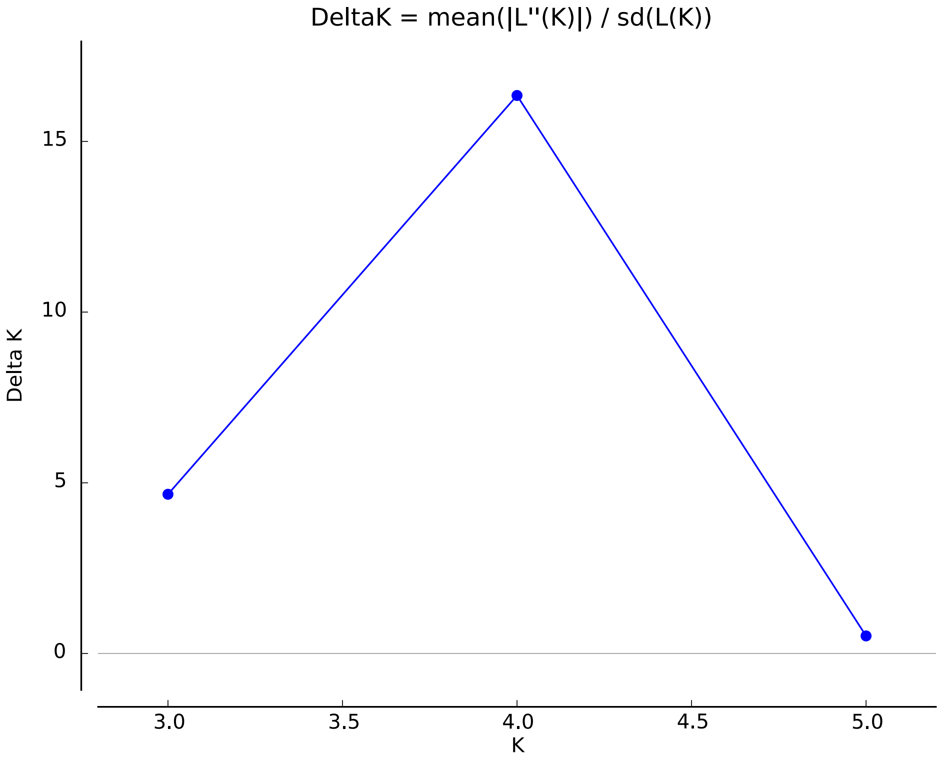

Table S1: Average genetic divergence among samples, F_{ST}, per locus

Locus	Cin-10B	Cin-12B	Cin-16B	Cin-1	Cin-13	Cin-15
Mean F_{ST}	0.093	0.060	0.055	0.060	0.091	0.054

Table S2: Multi-generation connectivity of *Ciona intestinalis* between three locations in the Kattegat-Skagerrak.

Connectivity was estimated with a biophysical model of larval dispersal in two depth intervals (0-12 m and 24-26 m) representing water masses above and below the pycnocline. Pelagic larval duration was set to 5 days and spawning time was set to May to September for larvae dispersing in shallow waters and June to July for larvae in deeper waters. The multi-generation, stepping-stone connectivity matrix over 32 generations, was calculated by multiplying the full dispersal matrix with itself 32 times and then extracting the connectivity between the three locations.

Drift depth: 0-12 m

	Väderöarna	Gullmarsfjorden	Vinga
Väderöarna	$1.3 \cdot 10^{-9}$	$6.0 \cdot 10^{-9}$	$1.4 \cdot 10^{-5}$
Gullmarsfjorden	$6.1 \cdot 10^{-9}$	$2.9 \cdot 10^{-8}$	$6.1 \cdot 10^{-5}$
Vinga	$2.9 \cdot 10^{-9}$	$1.4 \cdot 10^{-8}$	$3.9 \cdot 10^{-5}$

Drift depth: 24-26 m

	Väderöarna	Gullmarsfjorden	Vinga
Väderöarna	$3.6 \cdot 10^{-6}$	$3.2 \cdot 10^{-4}$	$7.9 \cdot 10^{-6}$
Gullmarsfjorden	$1.5 \cdot 10^{-5}$	$2.2 \cdot 10^{-3}$	$6.2 \cdot 10^{-6}$
Vinga	$1.9 \cdot 10^{-5}$	$1.2 \cdot 10^{-4}$	$1.2 \cdot 10^{-4}$

Table S3: Missing data and number of sites (out of 6) for which MICRO-CHECKER (van Oosterhout C et al. 2004) suggested null-alleles.

	Microsatellite locus					
	Cin-10B	Cin-12B	Cin-16B	Cin-1	Cin-13	Cin-15
Total N	240	240	240	240	240	240
Missing data	6	1	0	3	3	58
Null alleles?	6/6	0/6	3/6	3/6	6/6	6/6
F_{IS}	0.60	0.07	0.25	0.14	0.20	0.63
